# NADPH oxidases in Parkinson’s disease: a systematic review

**DOI:** 10.1186/s13024-017-0225-5

**Published:** 2017-11-13

**Authors:** Karim Belarbi, Elodie Cuvelier, Alain Destée, Bernard Gressier, Marie-Christine Chartier-Harlin

**Affiliations:** 1 University Lille, Inserm, CHU Lille, UMR-S 1172 - JPArc - Centre de Recherche Jean-Pierre AUBERT Neurosciences et Cancer, F-59000 Lille, France; 2 Inserm UMR S-1172 Team “Early stages of Parkinson’s Disease”, 1 Place de Verdun, 59006 Lille, France

**Keywords:** Alpha-synuclein, Microglia, Mitochondria, Neurodegenerative disorders, Oxidative stress, Synaptic plasticity

## Abstract

Parkinson’s disease (PD) is a progressive movement neurodegenerative disease associated with a loss of dopaminergic neurons in the substantia nigra of the brain. Oxidative stress, a condition that occurs due to imbalance in oxidant and antioxidant status, is thought to play an important role in dopaminergic neurotoxicity. Nicotinamide adenine dinucleotide phosphate (NADPH) oxidases are multi-subunit enzymatic complexes that generate reactive oxygen species as their primary function. Increased immunoreactivities for the NADPH oxidases catalytic subunits Nox1, Nox2 and Nox4 have been reported in the brain of PD patients. Furthermore, knockout or genetic inactivation of NADPH oxidases exert a neuroprotective effect and reduce detrimental aspects of pathology in experimental models of the disease. However, the connections between NADPH oxidases and the biological processes believed to contribute to neuronal death are not well known. This review provides a comprehensive summary of our current understanding about expression and physiological function of NADPH oxidases in neurons, microglia and astrocytes and their pathophysiological roles in PD. It summarizes the findings supporting the role of both microglial and neuronal NADPH oxidases in cellular disturbances associated with PD such as neuroinflammation, alpha-synuclein accumulation, mitochondrial and synaptic dysfunction or disruption of the autophagy-lysosome system. Furthermore, this review highlights different steps that are essential for NADPH oxidases enzymatic activity and pinpoints major obstacles to overcome for the development of effective NADPH oxidases inhibitors for PD.

## Background

Parkinson’s disease (PD) is the most prevalent movement disorder in elderly adults. It is characterized by the progressive degeneration of dopaminergic neurons in the *substantia nigra* and by the pathological accumulation of alpha-synuclein in protein aggregates named Lewy bodies in surviving neurons [1, 2]. Alpha-synuclein is a protein that is intracellularly localized in presynaptic terminals, where it has been shown to be involved in synaptic vesicle trafficking, synaptic function and plasticity [3]. However, alpha-synuclein can also be released from neurons and this process can be promoted under stress condition and enhanced by alpha-synuclein misfolding and aggregation [4, 5]. Sustained inflammation, defined by the presence of chronic microglial activation, is also consistently observed in the brain of patients [6]. These hallmarks are associated with dopamine deficit at the *striatum* -e.g. the striatal area innervated by the *substantia nigra*- that is the main factor leading to bradykinesia, resting tremor, rigidity and postural instability. It is generally accepted that these motor symptoms appear only after a substantial proportion of *substantia nigra* dopaminergic neurons are lost [7] suggesting that the disease has been engaged for years. This raises the possibility of adaptive or compensatory mechanisms in the early phase of this disease, involving synaptic plasticity of the remaining neurons [8, 9].

More than 90% of PD cases are sporadic and attributed to a combination of environment and/or genetic factors. Understanding the function of genes mutated in rare hereditary forms of PD has contributed to a better knowledge of PD pathogenesis. Missense mutations in the gene encoding alpha-synuclein *SNCA* (synuclein, alpha [non-A4 component of amyloid precursor]) were the first genetic abnormalities to be identified in PD families segregating as an autosomal dominant inherited trait [10]. Subsequently *SNCA* locus triplication and duplication were also shown as a cause of rare familial forms of PD [11–13]. Genetic evidence for a role of alpha-synuclein in sporadic PD emerged from the association between polymorphisms regulating *SNCA* levels and sporadic PD [14, 15], supporting that alpha-synuclein level is instrumental in the most common forms of the disease. Other mechanisms are involved. Mutations in genes encoding proteins of the endosomal/lysosomal system, vacuolar protein sorting-35 (*VPS35*), type 5 P-type ATPase ATP13A2, and glucocerebrosidase (*GBA1*) are also associated with PD [16] and represent a link between autophagy-lysosome function and neurodegeneration. Furthermore, overexpression of leucine-rich repeat kinase 2 (LRRK2), another PD-related protein, causes an increase in autophagosome numbers and lysosomal pH [17]. Other genes with a recessive PD-related inheritance such as *DJ-1, PARKIN,* PTEN-induced kinase 1 (*PINK1*) encode proteins playing an important role in the process of autophagy of mitochondria, known as mitophagy [16]. Mutations in one of these genes lead mitochondria to be morphologically aberrant and bioenergetically incompetent [18]. Although microglia activation and inflammatory changes are generally considered as a consequence of neuronal destruction, genome-wide analysis evidenced that the *HLA-DR* region [19] and that genes involved in the ‘regulation of leucocyte/lymphocyte activity’, ‘cytokine-mediated signaling’ and more generally in the immune system are associated with an increased susceptibility to PD [20, 21]. This raises the possibility that a general pro-inflammatory state could be a primary cause of neuronal loss in some cases or at least increases PD risk as a disease modifier genotype. Thus, the identification of these PD-related genes has led to the proposition that the progressive deterioration of dopaminergic neurons may arise from cellular disturbances produced by misfolding and aggregation of alpha-synuclein, mitochondrial dysfunction, disruption of the autophagy-lysosome system, endoplasmic reticulum stress, dysregulation of calcium homeostasis as well as chronic neuroinflammation [22, 23]. The discovery that environmental factors may be associated with PD promoted the creation of toxin-induced animal models designed to elucidate the mechanisms of neurodegeneration [24, 25]. Some of the most widely used toxins to study PD in animals include 6-hydroxydopamine (6-OHDA), 1-methyl-4-phenyl-1,2,3,6-tetrahydropyridine (MPTP) as well as pesticides such as rotenone (an insecticide) and paraquat (an herbicide). These models can present important features associated with the human disease including alpha-synuclein fibrillation, dopaminergic neuronal cell loss, mitochondrial dysfunction as well as oxidative damages (for review, see [26]).

The aforementioned cellular disturbances observed in the genetic and environmental models of PD are all closely linked to oxidative stress [23]. The term oxidative stress describes a redox imbalance between generations of free radicals or other reactive species and antioxidant defenses, and it may be related to changes in microglia activation, protein clearance, mitochondrial function and the autophagy-lysosome system. Oxidative stress has long been hypothesized to be central in sporadic PD pathogenesis. It was already proposed in 1990 that free radicals generated from oxidation reactions inappropriately oxidize macromolecules resulting in cellular dysfunction and, ultimately, in cell death [27]. This hypothesis is supported by several data. First, dopaminergic neurons of the *substantia nigra* are particularly sensitive to oxidative stress due to their high neuromelanin content, the generation and auto-oxidation of dopamine generate oxygen species. Second, this area is highly demanding in energy and the discovery in the early 1980s of the mechanism of toxicity of MPTP showed that inhibition of mitochondrial complex I activity causes a degeneration of the nigrostriatal neurons and a parkinsonian syndrome in humans, rodents or primates (as reviewed in [28]). Third, studies of postmortem brain tissues demonstrate increased oxidation of proteins [29], lipids [30] and DNA [31] and decreased levels of the antioxidant glutathione [32, 33] in the *substantia nigra* of PD patients. Finally, The *substantia nigra* contains the highest density of microglia in both human [34] and rodents [35] and their activation may therefore encounter an excessively high level of oxidative stress. Uncertainty about the molecular mechanisms leading to the oxidative stress in sporadic PD remains. A moderate deficit in mitochondrial complex I has been repeatedly evidenced in the *substantia nigra* of PD patients [36–38]. Furthermore, multiple aforementioned genes in which mutations or polymorphisms increase the risk of PD are linked to mitochondrial function or autophagy. As a consequence, it is considered that accumulation of bioenergetically compromised mitochondria could contribute to reactive oxygen generation in PD. However, other sources of reactive oxygen species in the nervous system could also be involved.

In the present review, we consider the role in PD of enzymatic sources that generate reactive oxygen species as their primary function that are the nicotinamide adenine dinucleotide phosphate (NADPH) oxidases. NADPH oxidases comprise a family of multi-subunit membrane-bound enzymes. Several NADPH oxidases have been evidenced in both neurons and glial cells in the brain where they contribute to a wide range of physiological functions related for example to host-defense or long-term synaptic plasticity. However, an overproduction of reactive oxygen species by NADPH oxidases could be detrimental. Based on the literature, we discuss how both microglial and neuronal NADPH oxidases could contribute to key cellular disturbances in PD such as microglia activation, alpha-synuclein accumulation, mitochondrial and synaptic dysfunction or disruption of the autophagy-lysosome system.

## The family of NADPH oxidases

NADPH oxidases are multi-subunit enzymes that primary catalytic function is the generation of reactive oxygen species. They function as electron transporters, using reduced NADPH as electron donor and molecular oxygen as electron acceptor to generate superoxide and/or hydrogen peroxide. Reactive oxygen species generation by NADPH oxidases was first discovered in polymorphonuclear neutrophils [39] as the enzyme responsible for the respiratory burst essential to the microbicidal function of these cells [40]. To the phagocytic catalytic subunit Gp91phox, called Nox2 (genomic location Xp21.1-p11.4) in the novel terminology, have been added six additional catalytic subunits of the same family: Nox1 (Xq22.1) [41, 42], Nox3 (6q25.3) [43], Nox4 (11q14.3) [44, 45], Nox5 (15q23) [46] and dual oxidase 1 and 2 (Duox1 15q21.1 and Duox2 15q21.10) [47, 48]. Duox1 and Duox2 are termed dual oxidase because they contain both a NADPH oxidase domain and a peroxidase-like domain. However, according to current knowledge, these two catalytic subunits do not display any peroxidase activity in human [49–51]. They are also named Nox6 and Nox7 according to [52].

**Fig. 1:**
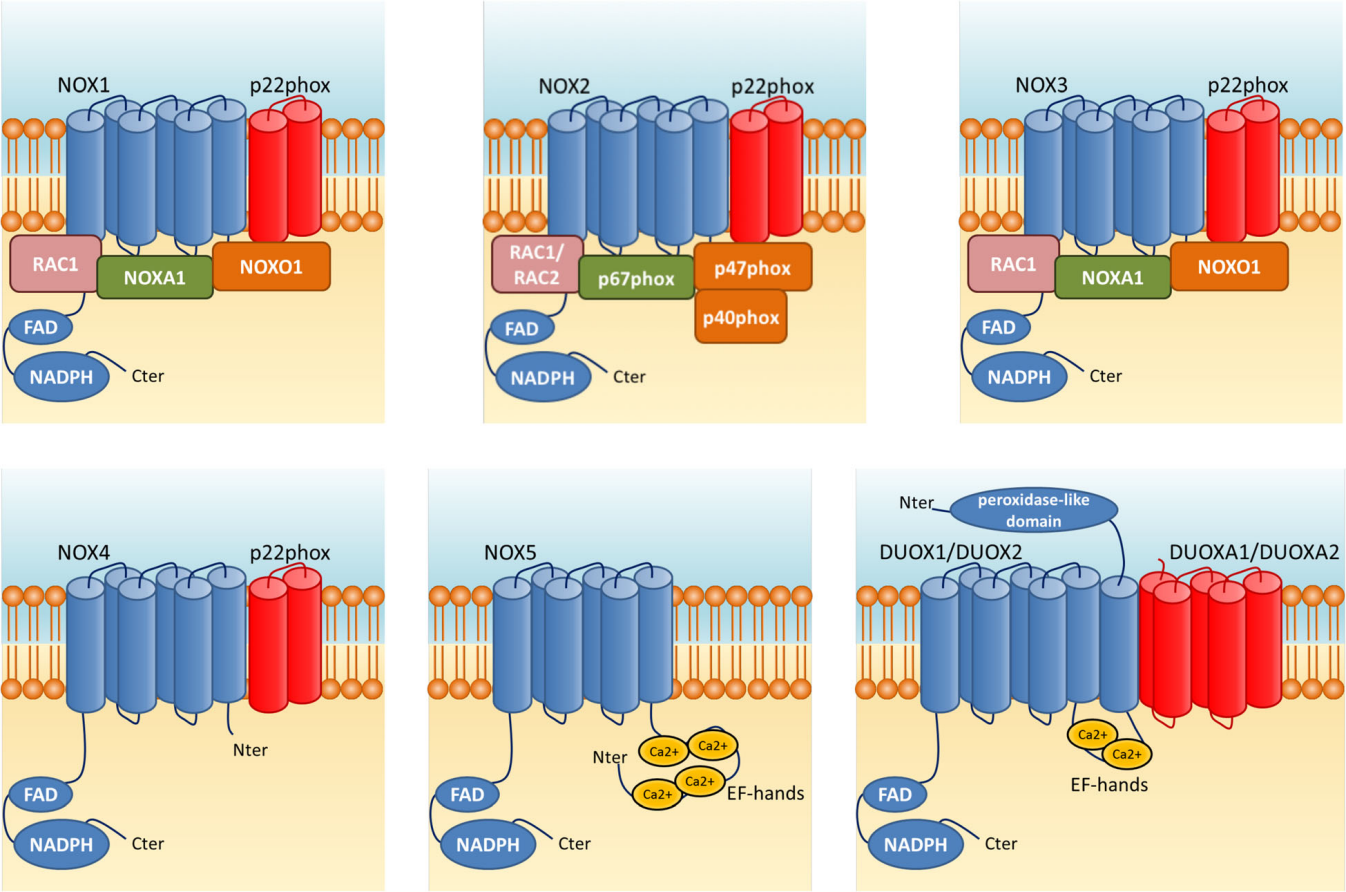
Activation of the NADPH oxidase family members. The figure illustrates for each NADPH oxidase the catalytic core region (in blue), the transmembrane maturation and stabilization subunits (in red) as well as the cytosolic subunits and the small GTPases (Rac1 and Rac2). The predicted regions for FAD and NADPH binding sites and the putative peroxidase-like region are also shown, as well as the EF hand motifs (yellow circles) that bind to Ca^2+^

All catalytic subunits of the NADPH oxidases family contain a ‘NADPH oxidase domain’ that is characterized by at least six membrane-spanning alpha-helical domains containing two hemes, a predicted region for flavin adenine dinucleotide (FAD) and a NADPH binding site in the cytosolic C-terminus [53] (Fig. 1). Despite this similar core structure, the NADPH oxidase family members differ in their subunit requirements (Table 1). Some of the NADPH oxidase catalytic subunits require association with other proteins that function as subunits such as p22phox, p40phox, p47phox, and p67phox and necessitate small GTPase Rac1 or Rac2 [54], as listed below. Nox1 requires both p22phox and NoxA1 for activation. The activation of Nox2, that is permanently membrane bound and associated with the p22phox subunit [55], needs the cytosolic subunits p40phox, p47phox, p67phox and the small GTPase Rac1 or Rac2 to migrate to the plasma membrane and assemble [56, 57]. In microglia, the phosphorylation and translocation of p47phox appears as the limiting factor for Nox2 activation [58] and various kinases have been implicated in p47phox phosphorylation. These include protein kinase C (PKC) isoforms [59–62], Akt [63, 64] mitogen-activated protein kinases (MAPK) [65], p21-activated kinase (Pak) [66] and extracellular signal-regulated kinase (ERK)1/2 [67]. Regulation of Nox3 appears to depend of the species and in human it needs activator subunits such as p22phox, NoxO1, NoxA1 and Rac for its activity. Nox4 requires p22phox [68] but does not involve cytosolic subunits. It is constitutively active in reconstituted systems [44, 69] and can be regulated in response to cytokines and growth factors such as insulin-like growth factor-I and transforming growth factor-β [70–72]. Nox5, Duox1 and Duox2 are activated by an elevation in intracellular Ca^2+^ and do not appear to require subunits, either membrane-bound or cytosolic [46, 73].

**Table 1:** Human NADPH oxidases genes, genomic positions and isoforms

NADPH oxidase catalytic subunit	Official Gene symbol and Full Name HUGO^a^		Other Proposed Aliases^b^	Entrez Gene IDd^b^	Genomic location^b^	mRNA (RefSeq Accession Numbers; GRCh38/hg38 human genome)^c^
Nox1	*NOX1*	NADPH oxidase 1	MOX1; NOH1; NOH-1; GP91-2	27035	Xq22.1	NM_007052 (isoform 1)
						NM_013955 (isoform 2)
						NM_001271815 (isoform 3)
Nox2	*CYBB*	cytochrome b-245 beta chain	CGD; NOX2; IMD34; AMCBX2; GP91-1; GP91PHOX; p91-PHOX; GP91-PHOX	1536	Xp21.1-p11.4	NM_000397 → NP_000388
Nox3	*NOX3*	NADPH oxidase 3	het; GP91-3; nmf250	50508	6q25.3	NM_015718 → NP_056533
Nox4	*NOX4*	NADPH oxidase 4	KOX; KOX-1; RENOX	50507	11q14.3	NM_016931 (isoform a)
						NM_001143836 (isoform b)
						NM_001143837 (isoform c)
						NM_001291926 (isoform d)
						NM_001291927 (isoform e)
						NM_001291929 (isoform f)
						NM_001300995 (isoform g)
						NR_120406
Nox5	*NOX5*	NADPH oxidase 5	NOX5A, NOX5B	79400	15q23	NM_024505 (isoform 1)
						NM_001184779 (isoform 2)
						NM_001184780 (isoform 3)
						NR_033671
						NR_033672
Duox1	*DUOX1*	dual oxidase 1	LNOX1; THOX1; NOXEF1	53905	15q21.1	NM_017434 (dual oxidase 1 precursor)
						NM_175940 (dual oxidase 1 precursor)
Duox2	*DUOX2*	dual oxidase 2	TDH6; LNOX2; THOX2; NOXEF2; P138-TOX	50506	15q21.1	NM_001190392 dual oxidase 2 precursor
						NM_177610 dual oxidase 2 precursor
						NM_213999 dual oxidase 2 precursor
						NM_024141 dual oxidase 2 precursor

^a^HUGO: http://www.genenames.org/. Accessed January 5 2017

^b^NCBI Entrez Gene: https://www.ncbi.nlm.nih.gov/gene/. Accessed January 5 2017

^c^human GRCh38/hg38 genome: https://genome.ucsc.edu/. Accessed January 5 2017

The patterns of activation of the different NADPH oxidase family members are summarized in Fig. 1. It should be noted that nearly all catalytic subunit display different transcripts and isoforms, and thus the properties of these different family members are probably more complex, possibly to enable specific and fine regulation processes (Table 1). Thus, the existence of various catalytic NADPH oxidases with differing activation properties and isoforms, together with various tissue and cell distribution, suggests that NADPH oxidases may have numerous biological functions.

## NADPH oxidases in the central nervous system

Since their identification in polymorphonuclear neutrophils, NADPH oxidases have been evidenced in non-phagocytic cells and in various organs [74], including the brain. A study showed that within total human brain mRNA, Nox2 is predominantly present together with traces of Nox4 and Nox5 transcripts [43]. Another study detected Nox1, Nox3 and Duox1 in the rat brain tissues [75], further confirming that NADPH oxidases are a plausible generators of reactive oxygen species in the brain.

**Fig. 2:**
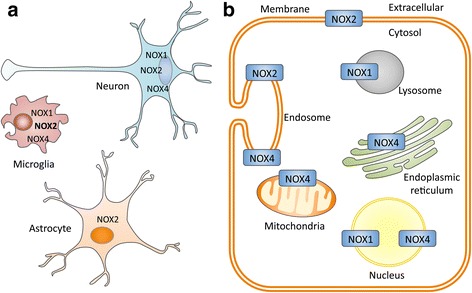
Cellular and subcellular expression of NADPH oxidase catalytic subunits in the brain. **a** Schematic diagram showing the reported cellular localization of NADPH oxidase family members in the brain cells. **b** Schematic diagram showing the reported subcellular localization of NADPH oxidase family members in a hypothetical cell in the brain

The distribution of NADPH oxidases in the brain has been studied at the cellular level, in microglia, the resident immune cells of the central nervous system, in neurons and astrocytes (supporting glial cells). In microglia of both humans and rodents, analyses showed that Nox2 is the main NADPH oxidase catalytic subunit present (Fig. 2). Lower levels of Nox1 and Nox4, but not Nox3, have also been documented in microglia at the transcript level [76–81]. In resting microglia and macrophages that can infiltrate the brain under certain pathological conditions, Nox2 localizes to the plasma membrane into cholesterol-enriched membrane microdomains (lipid rafts) together with p22phox [77, 82]. Following macrophage/microglia activation, it is internalized by clathrin-coated pits and redistributed to an intracellular compartment consisting of numerous small (<100 nm) vesicles [83]. Nox1 in microglia appears to localize in intracellular vesicular compartments including lysosomes, and can be recruited to phagosomal membranes [79].

Neurons have been shown to express Nox1 (mRNA; [84]), Nox2 (mRNA and protein; [85, 86]) and Nox4 (mRNA and protein; [87]). Nox4 is predominantly associated with the internal membranes including the endoplasmic reticulum and the endosomes and the mitochondria membrane [68, 88]. In PD patients, Nox1 and Nox4 have been observed in the nucleus of dopaminergic neurons [89, 90]. While present in most mammals, the Nox5 gene is absent from rodent genomes [53], therefore preventing the study of the endogenous protein in murine experimental models.

Finally, Nox2 appears to be the predominant NADPH oxidase family member expressed in astrocytes where its expression was reported at both the mRNA and protein level. In line, a significant decrease in reactive oxygen species generation is evidenced in astrocytes from Nox2-deficient mice [91, 92]. The cellular and subcellular expression of NADPH oxidases in the brain is summarized in Fig. 2.

The physiological functions of NADPH oxidase enzymes are various, including host defense and inflammation, post-translational processing of proteins, cellular signaling and regulation of gene expression (for review, see [74, 93]). The majority of publications on the physiological roles of NADPH oxidases in the central nervous system have focused on their participation in the host defense and the removal of debris from the brain. NADPH oxidases have also been shown to regulate neuronal cell fate and function and plasticity, for instance contributing to the induction of neuronal apoptosis in response to serum deprivation [85], to N-methyl-D-aspartate (NMDA) receptor signaling [94] and to long-term potentiation [95]. In the following paragraphs, we provide a thorough review of existing knowledge and information relative to the contribution of specific NADPH oxidase family members in microglia and neurons and highlight the main conclusions regarding PD pathogenesis.

## NADPH oxidases and microglia activation in PD

Microglia are the resident mononuclear phagocytes of the central nervous system, belonging to the glial system of non-neuronal cells that support and protect neuronal functions. Microglia comprise 5–20% of the total glial cell population within the central nervous system parenchyma and most densely populated areas include the *substantia nigra* [96]. Microglia are not only the first immune sentinels of infection, contributing to both innate and adaptive immune responses locally, but also play intrinsic functions required for normal brain function and neuroprotective effects. Chronic microglia activation is consistently reported in the pathogenesis of PD [97] and can be triggered experimentally by several PD-causing gene products such as alpha-synuclein or DJ-1.

### Microglial Nox2 expression is increased in PD and experimental models of PD

In 2003, Przedborski and coworkers reported that postmortem *substantia nigra* samples from sporadic PD patients had higher Nox2 (then referred as gp91phox) protein content than samples from control individuals (six PD patients versus three controls; mean duration of disease of 16.8 ± 2.3 years) [98]. Nox2 immunostaining localized with the microglial marker CD68, but not with neuromelanin. An increase in microglial Nox2 was also observed in microglia in the ventral midbrain of mice (brain region containing the *substantia nigra pars compacta*) after repeated intraperitoneal injections of MPTP [98]. In this model, the microglial expression of Nox2 was confirmed by immunostaining co-localization with the marker Macrophage antigen complex-1 (Mac-1) [98] and by ex vivo approaches showing an increase by approximately 3.5-fold in Nox2 expression in microglia acutely isolated, compared to saline-treated mice [99]. The MPTP model also show increased p67phox gene expression [98], translocation of the subunit p67phox from the cytosol to the plasma membrane [100], as well as induced p47phox phosphorylation and p47phox–Nox2 complexes in *substantia nigra* tissues [101], further confirming the activation of the enzyme. A marked increase in Nox2 protein level in reactive microglia was also documented in experimental models of PD based on combined administration of minimally toxic dose of LPS and alpha-synuclein oligomers [102], intraperitoneal injection of paraquat [103] or 6-OHDA [104, 105], on exposure to atmospheric ultrafine particles considered as a potential environmental risk factor for PD [106] as well as with aging and traumatic brain injury known to increase the risk of parkinsonism [107–109].

Taken together, these data strongly suggest that microglia are the predominant Nox2-expressing cells in PD and in several experimental models of PD and that Nox2 activation could contribute to its pathophysiology.

### Nox-2 modulates microglial responses and neurotoxicity

Microglial cells have a wide range of profiles and actions. On one hand, sustained classical microglia activation - consistently detected in the *substantia nigra* of patients with PD [6, 110–112] - could promote the slow degeneration of dopaminergic neurons. This is demonstrated in rodent models based on lipopolysaccharide administration [113, 114]. On the other hand, microglia act as neuroprotective cells through the elimination of endogenous or exogenous substances and participate to the resolution of the inflammatory response [115]. Moreover microglia have high levels of glutathione and glutathione peroxidase, which act to protect them and possibly neurons from toxic levels of H_2_O_2_ [116].

Lipopolysaccharide activates microglia primarily through the pattern recognition receptor Toll-like receptor (TLR) 4 signaling pathway [113, 117, 118]. However, lipopolysaccharide stimulation of Nox2 activity in microglia mainly occurs through binding of lipopolysaccharide to Mac-1, also named complement receptor (CR) 3 [119, 120]. Mac-1/CR3 is composed of CD11b (integrin αM) and CD18 (integrin β2) subunits. In Nox2-deficient mice, lipopolysaccharide fails to induce classical microglia activation as evaluated by Iba1 immunoreactive cells morphology, intensified F4/80 staining or induction of tumor necrosis factor (TNF)-alpha expression. Furthermore, lipopolysaccharide-induced loss of dopaminergic neurons is attenuated compared to Nox2+/+ mice [121, 122]. Also, Nox2 deficient microglia do not migrate towards substance P, a proinflammatory neuropeptide with high concentrations in the *substantia nigra* [123], supporting that Nox2 could be implicated both in the recruitment and the classical activation of microglia.

Nox2 could play a role in the deleterious effects of microglia in PD. For instance studies using cell culture systems revealed that microglia lacking functional Nox2 fail to produce neurotoxicity in response to MPTP [124, 125], paraquat [126] or rotenone in contrast to Nox2+/+ microglia [127]. This was corroborated by numerous in vivo studies showing that mice lacking Nox2 are less sensitive to dopaminergic degeneration induced by pesticides. For example, daily subcutaneous injections of MPTP results in a 32% loss of tyrosine-hydroxylase immunoreactive neurons in Nox2+/+ mice compared to a 14% loss in Nox2-deficient mice [128]. Similar attenuations of neurotoxicity in the Nox2−/− background were observed in mice receiving MPTP injections [98], paraquat injections [103] and in mice lesioned with 6-OHDA [104, 129]. Consistent with this protection, mice defective in Nox2 show less production of proinflammatory cytokines interleukin-1beta, TNF-alpha or interferon gamma (6-OHDA model; [104]) and less reactive oxygen species production and protein oxidation (MPTP model; [98]). Recently, Zhang and colleagues investigated the mechanisms underlying dopaminergic neurodegeneration using in vivo and in vitro models based on exposure to minimally toxic dose of LPS and alpha-synuclein oligomers. In their study, synergistic dopaminergic neurotoxicity - indicated by reduced dopamine uptake capacity, dopaminergic neuronal numbers in the *substantia nigra pars compacta* and depleted dopaminergic level in striatum – was reduced in Nox2−/− mice compared with Nox2+/+ mice [102]. Furthermore, microglial production of superoxides and reactive oxygen species were robustly reduced in Nox2−/− mice compared to Nox2+/+ mice. Thus, both exogenous and endogenous factors involved in PD appear to propagate classical microglia activation and dopaminergic neurodegeneration through activating microglial Nox2.

A role for Nox2 deficiency to promote neuroprotective role of microglia might emerge from the study of Hernandes and colleagues. In this study, Nox2−/− mice were treated with minocycline or saline and received 6-OHDA injections. Minocycline is a tetracycline derivative that exerts multiple anti-inflammatory effects, including microglial inhibition. Interestingly, the degeneration of dopaminergic neurons after 6-OHDA injections is greater in Nox2−/− mice that were treated with minocycline compared to Nox2−/− mice treated with vehicle [104]. Minocycline treatment also leads to NF-kappaB activation and increases TNF-alpha release into the *substantia nigra* of Nox2−/− 6-OHDA lesioned mice [104]. Therefore inhibiting Nox2−/− microglia cells likely increases *substantia nigra* degeneration and parkinsonism, suggesting a protective role for Nox2−/− microglia.

Although more studies are needed, these data demonstrate that Nox2 adds an essential level of regulation to signaling pathways underlying the inflammatory response. While microglial Nox2 appears to contribute to the chemoattraction, the classical activation and the toxicity towards dopaminergic neurons of microglial cells, inhibiting Nox2 signaling in microglia could favor their neuroprotective profile and actions in the context of PD.

### Alpha-synuclein, DJ-1 and Nox2 in microglia

Among the factors linked to the etiology of PD, alpha-synuclein and DJ-1 have been linked to microglia activation and could modulate NADPH oxidase activity in several ways as described below. Alpha-synuclein has a direct effect on microglial activation in vitro resulting in an overall increase in proinflammatory molecules and oxidative stress [130]. The signaling pathways mediating this process are multiple, and may depend of the structure and/or aggregation state of alpha-synuclein. For instance, alpha-synuclein monomers and fibrils were shown to induce interleukin-1beta release from monocytes via the TLR2 [131] and oligomeric forms of alpha-synuclein also specifically activate TLR2 [132]. However, the TLR4 has also been implicated in alpha-synuclein-induced inflammation [133]. A role for Mac1 has been proposed and for instance the binding of alpha-synuclein to Mac1 has been involved in oxidant release from microglia [134]. Finally, purinergic receptors have been implicated as a direct association between alpha-synuclein and the ionotropic P2X7 purinergic receptor leads to Nox2 activation through the phosphoinositide 3-kinases (PI3K) signaling pathway [135].

**Fig. 3:**
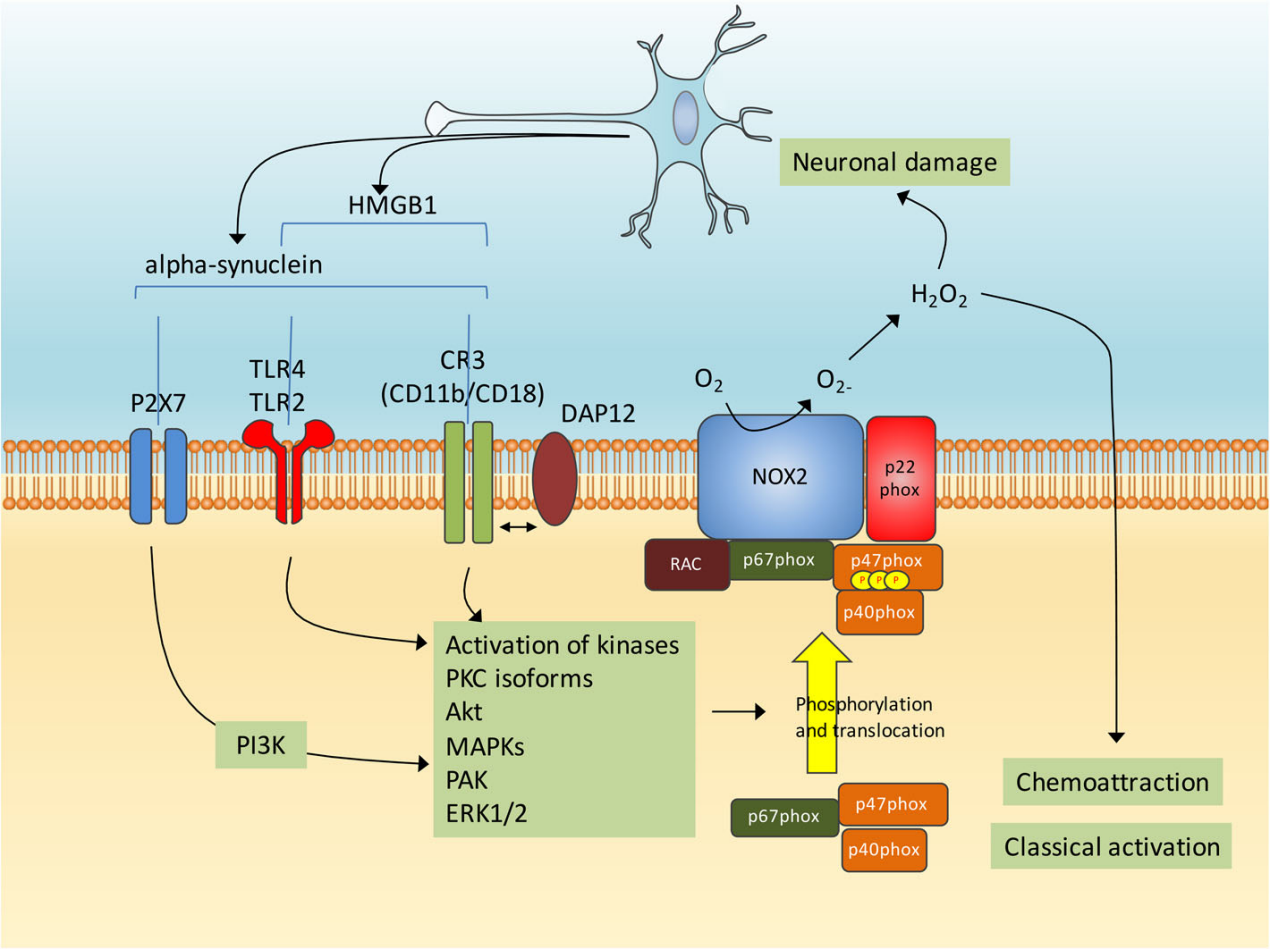
Alpha-synuclein and microglial Nox2 activation. The activation of microglia by alpha-synuclein can implicate several cell surface receptors such as P2X7, TLR2/4 and CR3 and subsequent activations of several kinases such as PKC, Akt, MAPKs, PAK and ERK1/2. This in turn could promote the phosphorylation and translocation of p47phox and subsequent Nox2 activation. Released oxygen species appear to promote microglia chemoattraction, activation and oxidative stress. Neuronal damage leads to the release of alpha-synuclein and the TLR-agonist high mobility group box protein 1 (HMGB1)

Both wild-type and A53T mutant alpha-synuclein were shown to activate Nox2 in BV2 microglial cells and in primary cultured microglia, with the A53T form producing quickest and sustained effects in terms of oxidative stress and cellular injuries. Interestingly, the process is partly blocked when BV2 cells are pretreated with LY294002, a strong inhibitor of PI3K [135]. Further supporting that alpha-synuclein promotes microglial Nox2 expression in pathological conditions, neuronal alpha-synuclein levels are elevated after spinal cord ischemic/reperfused injury and when cocultured with injured neurons or supernatants from injured neurons, Nox2 expression, reactive oxygen species generation and TNF-alpha expression are promoted in microglia. In this model, microglia activation is impeded by pretreatment with alpha-synuclein antibody or TLR2 antibodies and Nox2 levels in microglia are reduced by the pharmacological inhibition of MAPK p38 [136]. The comparison of the ability of various alpha-synuclein peptides to activate microglia allowed the identification of a specific peptide consisting of amino acids A29-V40 of alpha-synuclein that can directly bind to Nox2 resulting in NADPH oxidase complex activation [137]. When administered to wild-type mice, the A29-V40 peptide increases the expression of MHC-II, a cell surface marker of microglia classical activation [138], as well as the amount of malondialdehyde, one of the products during lipid peroxidation. In contrast, administration of the A29-V40 peptide has no such effects in Nox2−/− mice, suggesting that this alpha-synuclein peptide activates microglia and elicits oxidative stress in vivo in a Nox2-dependent manner [137]. Of note, a recent study indicates that the reactive oxygen species originating from activated Nox2 also serve as a direct signal driving microglial directional migration induced by the binding of alpha-synuclein to CD11b [139]. Thus, it is likely that several signaling pathways link alpha-synuclein with Nox2 and microglia activation (Fig. 3). Altogether, these findings show an important role for microglia Nox2 in mediating alpha-synuclein elicited microglia chemoattraction, activation and oxidative stress.

DJ-1 is another PD’s related gene product that has been suggested to have several possible functions including roles as an oxidative stress sensor, a protein chaperone, a protease, an RNA-binding protein, a transcription regulator, a regulator of mitochondria function and a regulator of autophagy. Recently, Liu and colleagues reported that DJ-1 binds to p47phox and that DJ-1 deficiency blunts TLR signaling and impairs NADPH oxidase-dependent reactive oxygen species production in macrophages. They moreover reported in DJ-1−/− versus control mice increased bacterial burdens, reduced local and systemic inflammation, macrophage paralysis and impaired induction of proinflammatory cytokines under the condition of sepsis. Importantly, in vivo administration of DJ-1 restored macrophages and rescued animals from septic death induced by lipopolysaccharide [140]. The study by Amatullah and colleagues also demonstrated the binding of DJ-1 with p47phox. In contrast with the aforementioned study, absence of DJ-1 resulted overall in increased reactive oxygen reactive species production and in increased NADPH oxidase activity, as observed in a mouse model of polymicroglial sepsis (the cecal ligation and puncture model) and in bone marrow-macrophages in response to LPS. The authors then demonstrated that DJ-1-p47phox interaction could disrupt the NADPH oxidase complex assembly and/or facilitate Nox2 ubiquitination and degradation thereby decreasing reactive oxygen species production [141]. Taken together, these two studies evidence that DJ-1 binds to p47phox and can modulate NADPH-oxidase function. Future studies are needed to precise the implication of this interaction regarding oxidative stress and inflammation PD and to determine whether PD-related DJ-1 mutations impact on this interaction.

Altogether, these data show that both alpha-synuclein and DJ-1 can direct NADPH-oxidases activation in microglia and such signaling could likely interfere with their neurotoxic and neuroprotective effects. The relationship between NADPH-oxidases and other genetic factors associated with PD will require further investigation. Of note, induction of colitis by dextran sulfate sodium leads to an earlier and severer colitismice paralleled by an increased phosphorylation of LRRK2 in p47phox mutated mice [142]. Addressing whether NADPH-oxidase contribute to changes in the immune response along with LRRK2 in PD will be of prime interest as both factors are expressed in immune cells [143] and appear to be important player in microglial activation and in alpha-synuclein-mediated microglial activation as well [144].

## Neuronal NADPH oxidases and neurodegeneration in PD

As discussed in introduction, defects in several cellular processes may push substantia nigra dopaminergic neurons towards cell death. These include oxidative damage, energy failure associated with mitochondrial dysfunction, altered glutamatergic neurotransmission and alterations in protein degradation efficacy. The finding that NADPH oxidases localize to specific subcellular organelles in neurons, such as the nucleus, the mitochondria and pre-synaptic sites [145–148] has raised the possibility that they could contribute to these defects, with implications for the development of neuroprotective therapies.

### NADPH oxidases, oxidative damage and alpha-synuclein accumulation in neurons

The presence of oxidative damage to the content of dopaminergic neurons has been consistently reported in the substantia nigra of PD patients. Suggesting that NADPH oxidases could play a role in these damages, a confocal microscopy imaging study evidenced Nox1 in the nucleus of dopaminergic neurons in the *substantia nigra* of postmortem brains of four PD patients while no signal was detected in those from control individuals [89]. Nox1 expression is also increased in the *substantia nigra* dopaminergic neurons in mice or rats in response to intraperitoneal injections of paraquat [149, 150] or striatal stereotaxic injection of 6-OHDA [89]. In these models, rise in Nox1 expression is associated with an increase in total and proteinase-K resistant alpha-synuclein levels, as well as with lipid peroxidation (paraquat model; [150]) and immunoreactivity for the DNA oxidative stress marker, 8-oxo-dG (6-OHDA model; [89]). Nox1 knockdown or Rac1 inhibition achieved by stereotaxic delivery of adeno-associated virus serotype 2 (AAV2) particles into the rat *substantia nigra* significantly reduces 6-OHDA-elicited immunostaining with 8-oxo-dG and dopaminergic neuronal loss [89]. The selective knockdown of Nox1 in the *substantia nigra* also largely attenuates the paraquat-mediated increase of total and proteinase K-resistant alpha-synuclein, oligomer-specific A11 immunoreactivity, oxidative stress and dopaminergic neuronal loss [150]. The Nox1/Rac1 complex was further analyzed in N27 rat dopaminergic cells in which (i) Nox1 tagged with EGFP translocates into the nucleus following 6-OHDA or rotenone treatments and (ii) Nox1 and GTP-bound activated Rac1 were detected by immunoprecipitation of nuclear extracts after a 24 h treatment with 6-OHDA [89, 151]. Therefore Nox1 translocation to the nucleus likely promotes subsequent Nox1/Rac1-derived superoxide generation responsible for oxidative damages to the neuron and this is associated with increased detrimental alpha-synuclein levels.

More recently, Nox4 immunoreactivity was noted in the nucleus of dopaminergic neurons in PD patients at Braak stage 6 [90], a stage with widespread alpha-synuclein accumulation [152]. Very interestingly, when looking at the first-affected nigral subregion nigrosome 1, the authors evidenced that the nuclear expression of Nox4 increases stepwise from age-matched controls (*n* = 7) to asymptomatic (*n* = 3) to clinically-confirmed PD patients (*n* = 5). Besides being correlated with negative clinical output, the elevated nuclear expression of Nox4 is also associated with oxidative damage to DNA, caspase-3-mediated cell loss and increased distribution of the angiotensin II type 1 (AT1) receptor in the nucleus. Because the activation of the AT1 receptor stimulates NADPH oxidase activity [153, 154] and because AT1 receptor increases with age and in response to 6-OHDA treatment [155], the authors propose that angiotensin II/AT1/Nox4 axis-mediated oxidative stress could contribute to damages in neurons in PD [90].

### Cross talk between mitochondria and NADPH oxidases in neurodegeneration

The regulation and maintenance of brain function requires high amount of energy, consuming ~20% of total body energy. Neurons depend primarily in oxidative phosphorylation to meet their energy demands, while glucose metabolism is directed towards the pentose phosphate pathway to generate NADPH. Mitochondrial dysfunction in PD is likely to contribute to energy failure and to the excess of reactive oxygen species and subsequent oxidative damages. Mitochondria and NADPH oxidases are both major sources of superoxide induction and several lines of evidence suggest that they might be considered along in PD. A first argument is that mitochondria can control the transcriptional activation of Nox1 as demonstrated in osteocarcinoma cells. In this model the inactivation of mitochondrial genes leads to the down-regulation of Nox1 expression. Conversely, increasing mitochondrial superoxide levels by exposing the cells to inhibitors of electron transport chain such as rotenone or antimycin increases the expression of Nox1 [147]. A molecular signaling link between mitochondria and Nox1 was further investigated for its contribution to superoxide production and apoptosis induced by serum withdrawal in human 293 T cells [156]. In this model serum withdrawal promotes the production of reactive oxygen species by stimulating both the mitochondria and Nox1. Mitochondria respond to serum deprivation within a few minutes. The mitochondria-generated reactive oxygen species stimulate PI3K that in turn induces the translocation of Rac1 to membrane fractions and the Rac1/Nox1 interaction, leading to sustained accumulation of reactive oxygen species [156]. Serum withdrawal-treated cells eventually loose their viability, which is prevented by blocking either the mitochondria-dependent induction of reactive oxygen species using rotenone or potassium cyanide or the PI3K/Rac1/Nox1 pathway using dominant negative mutants or small interfering RNAs [156]. Taken together, these data provide great evidence of a signaling link between the mitochondria and Nox1, which could be crucial for the sustained accumulation of reactive oxygen species and cell death processes in PD.

PINK1, which is linked to autosomal recessive familial PD, is a mitochondria-targeted serine/threonine kinase. It is well established that PINK1 protects neurons from oxidative stress [157] and in particular, loss of PINK1 function causes dysregulation of mitochondrial calcium handling, resulting in mitochondrial calcium overload which stimulates reactive oxygen species production. Of interest, Gandhi and coworkers demonstrated that reduction of NOX2 expression in PINK1 knockdown neuroblastoma cells significantly attenuates reactive oxygen species production [158], therefore evidencing a new signaling pathway at the crossroad between mitochondrial stress, oxidative stress and NADPH-oxidases.

More recently Nox4 was also directly associated to mitochondria as evidenced by confocal microscopy imaging showing its colocalization with the mitochondrial dye Mitotraker both in mice and in a murine catecholaminergic cells [88]. As investigated in this study, the expression level of Nox4 increases following angiotensin II exposure and knockdown of Nox4 achieved by adenoviral-encoded small interfering RNA significantly attenuates the angiotensin II-induced increase in mitochondrial-localized superoxide production [88]. Therefore it is likely that Nox4 contributes significantly to superoxide production at the mitochondria in response to angiotensin. Although more studies are needed, one can hypothesize that NADPH oxidases activation at the mitochondria could interact with elements of the electron transport chain within the mitochondria, thus indirectly initiating the production of mitochondrial superoxide [159, 160].

In conclusion, a cross talk between mitochondria and NADPH oxidases may represent a feed-forward vicious cycle of reactive oxygen species production that could contribute to oxidative damages and neurodegeneration in PD. NADPH oxidases-targeted antioxidants might break this vicious cycle, reducing NADPH-oxidase and limiting reactive oxygen species production by mitochondria.

### NADPH oxidases, synaptic signaling and excitotoxicity

Neurons utilize most of their energy at the synapse and an impairment of the ability of neurons to undergo synaptic plasticity is key in several theories explaining the onset and the progression of PD [161]. Dopaminergic denervation causes a profound network rearrangement, with the appearance of distinct forms of aberrant synaptic plasticity. Synaptic alterations in PD are also associated with abnormal expression or function of the NMDA receptor, a glutamate receptor and ion channel protein found in nerve cells [162]. Reactive oxygen species are required for NMDA receptor-dependent activation of ERK and long term potentiation [163], both of them being implicated in PD [164]. A specific role for NADPH oxidases at the synapse was initially suggested by Tejada-Simon and colleagues who reported that Nox2, p22phox, p40phox, p47phox, p67phox, and Rac are enriched in synaptoneurosome preparations from mouse hippocampal homogenates. Dual immunofluorescent labeling also show that 67phox or Nox2 colocalize with synaptophysin and synaptotagmin, respectively, confirming their localization at pre-synaptic sites [145]. Isolated synaptosomes have been shown to exhibit NADPH-dependent oxygen consumption and quantitative production of superoxide radicals and these are partially inhibited by the Nox2 inhibitor apocynin [165]. This production appears to be significant as NADPH oxidases rather than mitochondria are identified as the major reactive oxygen species source in isolated synaptosomes from mice, as assayed using spin trapping electron paramagnetic resonance spectroscopy [166]. Taken together, these studies clearly demonstrate the localization of functional NADPH oxidases at the synapse of neurons, making of NADPH oxidases prominent candidates as a source for reactive oxygen species for the control of synaptic neurotransmission.

In 2005, Kishida and colleagues reported that pharmacological inhibition of the NADPH oxidases using diphenylene iodonium or the lack of the p47phox subunit inhibit NMDA receptor-dependent ERK activation in hippocampal slices of mice [94], suggesting a direct role for NADPH oxidases in this signaling pathway. In line, pharmacological inhibition of NADPH-oxidases by diphenylene iodonium or apocynin or knockdown of Nox2 or p47phox in mice block NMDA receptor-dependent early-phase long-term potentiation in the hippocampus, leaving the basal synaptic transmission intact [95]. Corroborating these results, the rapid increase in superoxide production induced by NMDA receptor activation is blocked by the Nox2 inhibitor apocynin and in neurons lacking the p47phox subunit, both *in cellulo* and in vivo [167]. Therefore, Nox2 appears as the primary source of NMDA-induced superoxide production in neurons, being critical for NMDA receptor-related synaptic plasticity. Although these data were obtained in the hippocampus, mice lacking Nox2 present deficits not only in memory formation but also in the rotating rod and open field tests, suggesting a role for NADPH oxidases in synaptic function in several brain areas [95]. Finally, a role for Nox2 has been evidenced in the release of glutamate and dopamine after the administration of the NMDA receptor antagonist ketamine in mice [168]. Future research is needed to explore whether NADPH oxidases deregulations could control NMDA receptor–dependent glutamate and dopamine release in the context of PD.

While NMDA receptor is critical for synaptic plasticity, its sustained activation leads to extensive superoxide production promoting neuronal death [169]. As such, the finding that NMDA receptor stimulation triggers NADPH oxidases activation also provides a mechanistic link between oxidative stress and excitotoxicity. Of interest, PI3K is essential for NMDA receptor-dependent activation of Nox2; for example the PI3K inhibitor wortmannin reduces NMDA-induced Nox2 activation and cell death in primary neuronal cultures [170]. Perturbations in the PI3K/Akt pathway have been reported in PD patients [171, 172] and, as such, may either increase or decrease Nox2 superoxide production thereby impacting synaptic plasticity, the release of neurotransmitters and excitotoxicity in PD. This suggests that deregulation in NADPH oxidases expression, localization or activation could directly contribute to synaptic defects and excitotoxicity in PD.

### NADPH oxidases and the autophagy-lysosome system

Autophagy is a dynamic cellular pathway involved in the degradation of misfolded proteins and other cellular constituents. Impairment of the autophagic flux has been evidenced in PD and could promote the accumulation of compromised mitochondria and alpha-synuclein [23]. Several publications reported that exposure to rotenone can impair the autophagic flux, resulting in cytosolic accumulation of the autophagosomal membrane form of microtubule-associated protein 1 light chain 3 (LC3) [173], increased p62 levels [174] and aberrant accumulation of alpha-synuclein [175]. This rotenone’s impact on autophagy likely occurs through the PI3K/Akt/mTOR signaling pathway [173, 176]. To determine whether rotenone-induced autophagy was Nox2 dependent, Pal and colleagues used the human neuroblastoma SH-SY5Y cell line. They observed that short exposure to rotenone (0.5 μM; 6 h) results in a ~2-fold increase in reactive oxygen species generation, impairs autophagic flux and promotes protein accumulation. Pre-incubation with the Nox2 docking sequence (Nox2ds)-tat significantly attenuates the levels of LC3 and p62 proteins, indicating that the effect on autophagy is mediated by Nox-2 dependent reactive oxygen species. When SH-SY5Y dopaminergic cells are exposed to higher doses of rotenone (10 μM; 24 h), a ~3.5 increase in reactive oxygen species generation is observed compared to untreated cells. Nox2-ds abolish Nox2-generated reactive oxygen species generation (measured using the Nox2-specific redox sensor p47-roGFP) while total intracellular reactive oxygen species (measured using DCF-DA) is partially, but not completely, inhibited [177]. This suggests that 10 μM rotenone for 24 h stimulates reactive oxygen species generation not only through Nox2, but also possibly from mitochondria. Importantly, preincubation with Nox2-ds partially attenuates LC3 and p62 protein levels and protects against rotenone-dependent upregulation in apoptotic signaling [177]. These data highlight a novel mechanism by which Nox2-dependent oxidative stress could promote the pathogenesis of PD. Noteworthy, Nox4 was also found to promote autophagy and survival in cancer cells [178] and in cardiomyocytes in response to nutrient deprivation and ischemia [179], while no studies have been performed yet in the neuronal context. Thus, this emerging evidence indicates the importance of NADPH oxidases in the regulation of autophagy. Future studies are warranted to delineate the association between NADPH oxidases-dependent impaired autophagy, mitochondria dysfunction and cell death in PD.

## NADPH oxidases inhibitors as potential therapeutic agents in PD

**Fig. 4:**
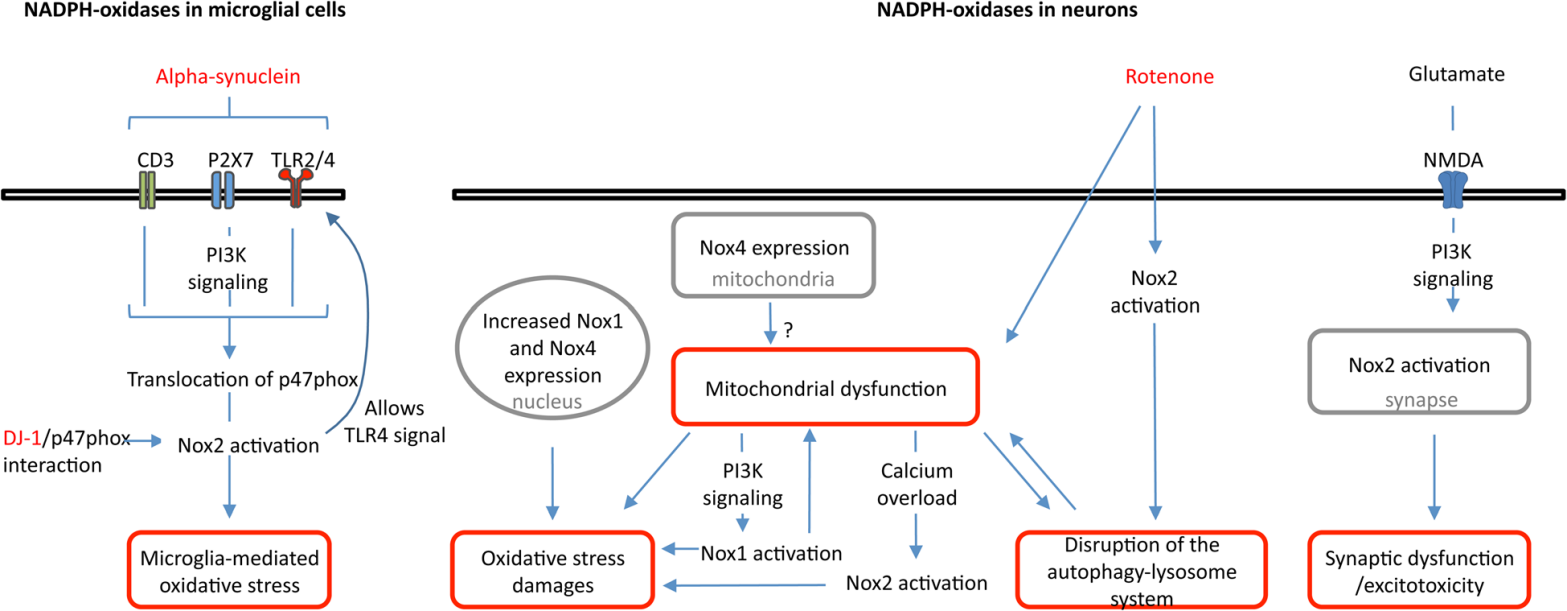
Proposed role for NADPH oxidases in PD. Schematic view of the link between both microglial and neuronal NADPH oxidases and cellular processes related to PD, e.g. alpha-synuclein signaling, microglia activation, oxidative stress and neuronal damage, mitochondria dysfunction, disruption of the autophagy-lysosome system, synaptic dysfunction and excitotoxicity. Localizations of NADPH oxidases are indicated in grey

To date, strategies directly targeting oxidative stress in PD have been typically focused on compounds that scavenge reactive oxygen species after they have been produced and on mitochondria-targeting therapeutics. Despite the importance of oxidative stress and mitochondria in PD, these strategies have shown mitigated or no success in clinical trials [180]. Modulating NADPH oxidases activity would represent a logical alternative to modify the course of PD, as it targets enzymatic complexes that are solely dedicated to the production of reactive oxygen species and that regulate cellular processes that are disrupted in PD (Fig. 4).

Different steps that are essential for NADPH oxidases enzymatic activity can be targeted. These include (i) the expression of NADPH oxidases subunits, (ii) the ligand-receptor binding, (iii) the complex assembly and its activation (including inhibiting signaling pathways such as PI3K signaling) as well as (iv) electron transfer. As illustrated in Table 2, several of these targets have been partially evaluated using either in vitro or in vivo experimental models of PD. These preclinical studies appear overall promising with for example decreases in markers of oxidative stress, in markers of autophagy-lysosomal defects or in neurodegeneration (Table 2). Beside knock-out mouse models and interfering RNAs, several small compounds or antibodies and peptides have been postulated as NADPH oxidases inhibitors. For a critical overview of the NADPH oxidases inhibitors, we refer the reader to the review of Altenhöfer and colleagues [52]. Briefly, the historical Nox2 inhibitors diphenylene iodonium and apocynin are commonly used but are unspecific and their effects cannot be solely attributed to inhibition of Nox2 or even NADPH oxidases [181]. More recently, several compounds inhibitors have been identified by rational drug discovery approaches and characterized with regards to NADPH oxidases selectivity and potential off-target effects. For example, ML171 is a potent Nox1 inhibitor with IC_50_ value of 130-150 nM for Nox1 and of 3-5 μM for Nox2-4. GKT136901 [182, 183] and GKT137831 [184] are two structurally related compounds developed by Genkyotex. They potently inhibit Nox1, Nox4 and Nox5 with ~10 lower IC_50_ value than for Nox2 inhibition. They are orally bio-available and have favorable ADME profiles [183, 184] and have consequently been evaluated across a range of disease models. GKT137831 has progressed through preclinical development and into clinical trials and thus appears as a good pharmacological tool for in vivo studies on the role of Nox1, Nox4 and Nox5 NADPH-oxidases enzymes in disease [185]. Peptide-based inhibitors such as Nox2-ds-tat, by their nature, have the potential advantage of being more specific and having fewer off-target effects than small-molecule organic compounds. However these peptides have disadvantages as well, such as low bioavailability and metabolic liability and - over time - induction of neutralizing antibodies. Overall, the high degree of structural and catalytic homology between the different catalytic subunits of NADPH oxidases makes finding selective inhibitors a challenge. However, as proposed by Diebold and collaborators several protein-protein interactions could in principle be targeted with new high-throughput assays. For example, targeting the binding of p67phox to p47phox or the binding of p47 to the membrane are suggested specific approaches to block the assembly of the active complex of Nox2 [186].

**Table 2:** Therapeutic targeting of NADPH oxidases in selected studies

NADPH oxidase signaling step	Mode of action	Inhibitors	Experimental model	Effects of the inhibitor on the experimental model	Current therapeutic roadblocks	Reference
Subunit expression	Decrease specific subunit mRNA and protein levels	Adeno-associated virus serotype 2 (AAV2) expression cassettes with Nox1shRNA	Striatal injection of 6-OHDA in the rat	Nox1 knockdown reduced 6-OHDA-induced oxidative DNA damage and dopaminergic neuronal degeneration.	Drug delivery	[89]
		Knockdown of Nox4 achieved by adenoviral-encoded small interfering RNA	Mouse catecholaminergic neuronal cell model (CATH.a) exposed to angiotensine II	Nox4 knockdown attenuated of angiotensine II-induced mitochondrial O_2_∙-production.	Drug delivery	[88]
Ligand-receptor binding	Block the interaction between alpha-synuclein and P2X7R	Brilliant Blue G, a P2X7R antagonist	BV2 microglial cells treated with wild type or A53T alpha-synuclein	Pretreatment with Brilliant Blue G reduced the translocation of p47phox from the cytoplasm to the membrane after treatment with each form of alpha-synuclein.	Targets one of many NADPH oxidase activators	[135]
Complex assembly and activation		AAV2 expression cassettes with a T17N dominant negative Rac1 variant	Striatal injection of 6-OHDA in the rat	Rac1 inhibition reduced 6-OHDA-induced oxidative DNA damage and dopaminergic neuronal degeneration.	Drug delivery	[89]
	Prevent p47phox association with NADPH oxidase complex	Apocynin	Lipopolysaccharide induced PD model: single injection of lipopolysaccharide at a dose of 5ug/5ul PBS into the SN of rats.	Apocynin prevents α-synuclein aggregation, microglial activation, dopaminergic neurodegeneration and relieves motor system abnormality following lipopolysaccharide injection.	Unspecific, dosing:	[189]
		gp91-ds,a peptide inhibitor for NADPH oxidase assembly	SH-SY5Y dopaminergic cells exposed to rotenone	Preincubation with Nox2-ds partially attenuated LC3 and p62 protein levels and protected against rotenone-dependent upregulation in apoptotic signaling (Pal et al., 2016).	Drug delivery, pharmacokinetic and CNS biodisponibility	[177]
	Block PI3K signaling needed for NADPH oxidase activation	LY294002, a potent and specific cell-permeable inhibitor of PI3K				[135]
Electron transfer	Extracts electrons	Diphenyleneiodonium	Neuron-glia cultures pretreated with lipopolysaccharide, 1-methyl-4-phenylpyridinium or rotenone	Diphenyleneiodonium protected the dopaminergic neurons at subpicomolar concentrations.	^2^non-specificity for other flavoenzymes and high cytotoxicity at standard doses (μM)	[190]

Beside the roadblocks having to do with delivery, pharmacokinetic, biodisponibility and specificity, the use of NADPH oxidases inhibitors will necessitate to determine the percent inhibition that could modify PD development while preserving physiological activity. NADPH oxidases have essential functions in host defense, regulation of cell growth and differentiation, regulation of vascular tone and of blood pressure [187], regulation of renal function [188] and, as broached in this review, in normal functioning of neurons. As such, excessive inhibition of NADPH oxidases could contribute to increased risk for infections, autoimmune disorders and/or tumor development, as well as cardiovascular diseases for example. Determining the therapeutic window for efficacy with minimal side effects is therefore needed for the acceptance of NADPH oxidases inhibitors as therapeutic agents in PD.

## Conclusions

Data are already available suggesting pathologically relevant implications of NADPH oxidases in PD. Due to overlapping expression of Nox1, Nox2, Nox4 in microglia, and neurons, it remains difficult to dissect the relative involvement of the different NADPH oxidases family members in the dopaminergic degeneration. Development of microglia or neuron-selective gene knockout models or functional rescue experiments in a constitutive knockout background may resolve this issue in the future. In parallel, identification through rational drug discovery approaches of inhibitors specific for a given NADPH oxidase is needed to further demonstrate the therapeutic potential of NADPH oxidases in PD. Nox2 potentiates microglia proinflammatory phenotype and its overactivation is observed both in patients and in response to several toxins associated with parkinsonism. Because P47phox (i) appears as the rate-limiting factor for Nox2 activation, (ii) directly binds to DJ-1 and (iii) is phosphorylated by several kinases activated downstream of pathways activated by alpha-synuclein, it is a potentially important target for the development of therapeutic agents against PD. The PI3K/Akt/mTOR pathway that is deregulated in PD and that promotes NADPH oxidases activity also appears as a signaling pathway to be considered for the development of therapeutic strategies. The neuronal Nox1 and Nox4 that are upregulated in dopaminergic neurons of patients are also of prime interest although more studies are needed to evaluate the role of other NADPH oxidases in dopaminergic degeneration and to potentially identify isoform-specific pathways. Of importance, the evaluation of NADPH oxidases targeting therapeutics should be based not only on the detection of reactive oxygen species and oxidative damages, but also in regards to the energy failure associated with mitochondria dysfunction, the synaptic dysfunction and the disruption of the autophagy-lysosome associated with PD.

## Abbreviations

6-OHDA: 6-hydroxydopamine
AAV2: Adeno-associated virus serotype 2
ERK: Extracellular signal-regulated kinase
FAD: Flavin adenine dinucleotide
HMGB1: High mobility group box protein 1
LC3: Microtubule-associated protein 1A/1B-light chain 3
MAC-1: Macrophage antigen complex-1
MAPK: Mitogen-activated protein kinases
MPTP: 1-Methyl-4-phenyl-1,2,3,6-tetrahydropyridine
mTOR: Mammalian target of rapamycin
NADPH: Nicotinamide adenine dinucleotide phosphate
NMDA: N-methyl-D-aspartate
PAK: P21-activated kinase
PD: Parkinson’s disease
PI3K: Phosphoinositide 3-kinases
PINK1: PTEN-induced kinase 1; Toll-like receptor
TNF: Tumor necrosis factor
